# Knowledge, attitude, and practice associated with antimicrobial resistance among medical students between 2017 and 2022: A survey in East China

**DOI:** 10.3389/fpubh.2022.1010582

**Published:** 2022-10-24

**Authors:** Shengyi Min, Yuxuan Zhou, Yuxuan Sun, Jiaquan Ye, Yongfei Dong, Xichao Wang, Zhemin Zhou, Hanyu Zhou, Heng Li

**Affiliations:** ^1^Pasteurien College, Suzhou Medical College, Soochow University, Suzhou, China; ^2^Department of Biostatistics, School of Public Health, Medical College of Soochow University, Suzhou, China; ^3^Jiangsu Key Laboratory of Preventive and Translational Medicine for Geriatric Diseases, Medical College of Soochow University, Suzhou, China; ^4^Central Laboratory, The First Affiliated Hospital of Wannan Medical College, Wuhu, China

**Keywords:** antimicrobial resistance, medical education, antimicrobial use, knowledge, attitude and practice study, AMR awareness

## Abstract

This study described the knowledge, attitude, practice regarding antimicrobial resistance (AMR) among medical students between 2017 and 2022 in East China. A questionnaire-based survey was conducted with a total of 1,066 respondents. We highlighted that the undergraduates had a significant increase in the knowledge of antimicrobial resistance during the 5 years from 2017 to 2022 (*p* < 0.001). The majority of the assertions about the AMR were correctly identified by respondents. However, gaps were still observed in the issues of antimicrobial targets and bacterial transmission. In addition, overconfident attitudes and inappropriate behaviors of antimicrobial overuse and misuse were observed in the respondents. A number of 30.2% to 45.2% of the respondents asserted that there is no risk of AMR as long as the antimicrobials are taken correctly, and a proportion of the students (25.3% in 2022; 69.3% in 2017, *p* < 0.001) declared to buy antimicrobials from friends or family members to treat the same illness. Finally, spearman correlation coefficient was enrolled to compare the correlation of the student's KAP. Results showed that the students' knowledge of antimicrobials had a correlation with attitude (*p* = 0.0126) and practice (*p* < 0.001), suggesting that public education on knowledge could influence the behaviors among the medical students. Taken all together, our findings show a need to strengthen the medical students' cogitation on antimicrobial attitude and practice of appropriate usage as an essential strategy to reduce intractable public health problems. Additional curriculum reforms will be needed to add more specific AMR-related lectures to raise awareness amongst medical students in China.

## Introduction

Antimicrobial resistance (AMR) has emerged as one of the most pressing challenges over the last two decades (1). World Health Organization endorsed a global action plan in May 2015 to provide safer antimicrobials for the prevention and treatment of infectious diseases (2). However, AMR continues to spread in many parts of the world, including developing countries such as China, Pakistan, and India (3–6). As one of the world's largest producers and consumers of antimicrobials, the Chinese government has implemented a number of policy measures e.g., the “*National Action Plan to Tackle Bacterial Resistance (NAPCBR, 2016–2020)*,” to address antimicrobial education in hospitals, community clinics, agricultural industry, and medical universities (7).

The purchase of antimicrobials is regulated by well-established legislation in China that antimicrobials can only be dispensed under the formal prescription from a medical practitioner (8). However, multidrug-resistant and extensively drug-resistant (XDR) bacterial strains were reported in the hospital (9). According to the China Antimicrobial Resistance Surveillance Trial Program (https://www.chinets.com), the isolation frequency of carbapenem-resistant *Klebsiella pneumoniae* had rapidly climbed from 2.9% in 2005 to 24.4% in 2021 (10). Additionally, extended-spectrum methicillin-resistant *Staphylococcus aureus* was detected in China and other countries as the emerging threat to public health (11–13).

To address the issues of AMR, previous studies have concentrated on the gap in knowledge, attitude, and practice (KAP) regarding antimicrobials among students (14–17). Previously, researchers investigated the KAP of Chinese undergraduates and discovered a large deficit in the knowledge section addressing the appropriate use of antimicrobials (15). The medical students' knowledge and attitude also drive their doctors' self-prescription of unneeded antimicrobials (18). Thus, advising future doctors and medical students on antimicrobial usage and dosage constitutes an important strategy to reduce bacterial drug resistance.

The Chinese *NAPCBR* (*2016–2020*) has counseled the public on appropriate AMR issues and antimicrobial prescribing for many years (7). To evaluate the awareness of antimicrobial resistance in medical education during the past 5-year, this study was conducted to investigate the KAP of medical students between 2017 and 2022. We hypothesized that the years of action on the necessity of antimicrobials had aided in raising awareness amongst medical students in East China.

## Method

### Respondents and study setting

This survey was questionnaire-based and aimed to assess the awareness of KAP among Chinese medical undergraduates between 2017 and 2022 in Yangtze River Delta region in eastern China. We selected one representative medical university in Suzhou as the research object. The sample size was measured by the online sample size calculator (https://www.calculator.net/) with a 50% population proportion, 5% margin of error, and 95% confidence level. The questionnaire was delivered to the students *via* Tencent forms on social media applications. The students were advised to fill out the questionnaire by clicking the link. A total of 1066 respondents were enrolled in this study with a response rate of 88.83% (1,066/1,200). The first round of the survey obtained 564 respondents during August and November 2017. The second round collected 502 feedbacks between August and October 2022 with either online or face-to-face interviews. All the respondents joined in with no incentives and signed the informed consents. Detailed characteristics were described in Table 1.

**Table 1:** Demographic characteristics of the respondents.

**Characteristics**	**Numbers (%)** ^*^	
**Year**	**2022**	**2017**
**Gender**
Female	297 (59.2%)	357 (63.3%)
Male	205 (40.8%)	207 (36.7%)
**Age group in years**
18~20	224 (44.6%)	211 (37.4%)
21~23	213 (42.4%)	256 (45.4%)
24~25	65 (13.0%)	97 (17.2%)
Total	502	564

* Numbers in parentheses are the percentage of respondents for each category (gender or age).

### Questionnaire design

A structured questionnaire was prepared by reviewing questionnaires of validated surveys that were previously reported and was customized in such a way to reflect issues relevant to China (14–17). The questionnaire was divided into five sections with subdivided components. The first section was basic information about the students, gender, age and education level. The second section contained two multiple choices on the knowledge of AMR. The third section included eight questions of knowledge which were validated by WHO report and peer studies (2, 15). The fourth section recorded 12 questions which were set in the part of attitude relating to the severity of antimicrobial abuse in healthcare and the impact and responsibility of reduction based on the validated surveys used in both China and other countries (15, 17, 19–21). Finally, the fifth section of the questionnaire included five questions regarding the practices of antimicrobial overuse, misuse, and AMR transmission (Supplementary materials-questionnaire) (15, 17, 21). Questionnaire validation was ensured by an expert team (2 professors and 2 physicians) and 5 undergraduate medical students. The questionnaire was translated into Chinese and improved the accuracy on advice of relevant experts from the field of statistics and epidemiology before being finalized.

### Grading standards

Grading standards were used to give each question specific scores. Standard answers such as “true” to “false” and “agree” to “disagree” were simultaneously determined corresponding to the suggestions from WHO (21). The grading standards were validated by similar surveys that were previously used in other research (15, 20). Specifically, 3 or 0 points were graded to the single or multiple-choice options and 1 to 5 points were allocated for five-answer responses as agree, agree slightly, neutral, disagree slightly, and disagree, respectively. All the questions and grading standards are presented in Supplementary materials-grading standard.

### Statistical analysis

**Figure 1:**
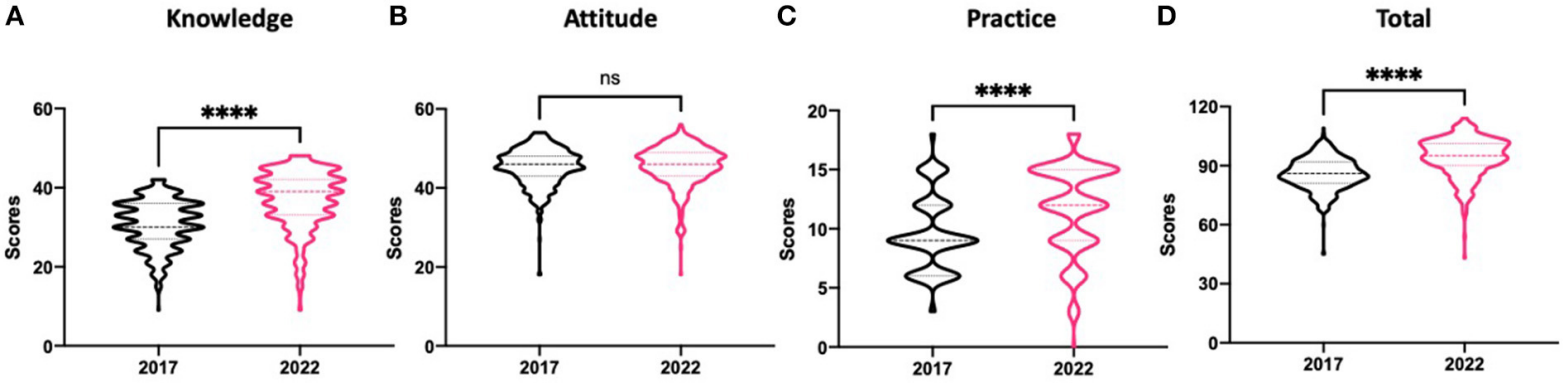
The grading graphs of respondents' knowledge, attitude, and practice on antimicrobials. Scores were calculated according to the grading standards and statistical test by Mann–Whitney with *p*-value < 0.001 (****) was considered statistically significant. Significances were observed in the scores of knowledge, practices and total respondents. **(A)** Knowledge scores were calculated based on the questions in Table 2A. Significances were observed among the respondents between 2017 and 2022 (*p* < 0.001). **(B)** Attitude scores were analyzed by evaluating the questions in Tables 2B and 3. No differences were found between the two groups (*p* = 0.2805). **(C)** Practice scores were computed based on the questions in Table 2C with significant differences detected (*p* < 0.001). **(D)** Total scores were statistically counted according to all the questions in Tables 2 and 3. Significances were observed among the two groups with regard to antimicrobial resistance (*p* < 0.001).

The grading scores were typed into SPSS 26.0 (IBM SPSS Statistics 26.0) and Graphpad prism 7 for statistical analysis (GraphPad Software Inc., San Diego, CA). For the data type of frequencies and percentages, descriptive statistics were used and were presented in tables. The median and IQR (Interquartile range defines the middle 50% of values from “lowest to highest” when ordered) were used to describe the data of attitude as appropriate. The difference of scores was determined through the Mann–Whitney statistical tests with *p* < 0.001 considered statistically significant (Figure 1). The categorical variables (such as the years) were done using Chi-square test to assess the association among these students toward antimicrobials (Table 2). Then rank sum test was enrolled for the analysis *via* Mann-Whitney *U*-test (Table 3). Finally, Spearman correlation coefficient was enrolled to compare the correlation of the student's knowledge, attitudes and practices between 2017 and 2022 with *p* < 0.001 as significant.

**Table 2:** The knowledge, attitude and practice of respondents regarding antimicrobials.

**Choice question (response)**	**Correct rate % (n/N)** ^*^		**χ^2^**	***P*-value**
	**2022**	**2017**		
**A. Knowledge**				
Q1. Antimicrobial resistance occurs when your body becomes resistant to antimicrobials and they no longer work as well (False).	67.3 (338/502)	45.6 (257/564)	51.010	<0.001
Q2. Many infections are becoming increasingly resistant to treatment by antimicrobials (True).	89.4 (449/502)	82.4 (465/564)	10.631	0.001
Q3. If bacteria are resistant to antimicrobials, it can be very difficult or impossible to treat the infections they cause (True).	85.1 (427/502)	80.7 (455/564)	3.578	0.059
Q4. Antimicrobial resistance is an issue that could affect me or my family (True).	89.4 (449/502)	88.5 (499/564)	0.252	0.615
Q5. Antimicrobial resistance is an issue in other countries but not here (False).	93.5 (469/502)	94.5 (533/564)	0.546	0.460
Q6. Antimicrobial resistance is only a problem for people who take antimicrobials regularly (False).	91.8 (461/502)	87.2 (492/564)	5.927	0.015
Q7. Bacteria which are resistant to antimicrobials can be spread from person to person (True).	76.5 (384/502)	77.8 (439/564)	0.272	0.602
Q8. Antimicrobial-resistant infections could make medical procedures like surgery, organ transplants and cancer treatment much more dangerous (True).	92.4 (464/502)	88.7 (500/564)	4.381	0.036
**B. Attitude**				
Q1. Do you think there is abuse of the current antimicrobials? (Yes)	66.5 (334/502)	73.4 (414/564)	5.989	0.014
Q2. Do you think antimicrobials are widely used in agriculture (including in food-producing animals) in your country? (Yes)	44.6 (224/502)	77.5 (437/564)	121.741	<0.001
**C. Practice**				
Q1. Where do the antimicrobials you used come from? (doctor or nurse)	80.9 (406/502)	77.1 (435/564)	2.242	0.134
Q2. Will you ask the doctor to prescribe for you antimicrobials when you catch a common cold? (Yes, I will.)	4.8 (24/502)	6.2 (35/564)	1.031	0.310
Q3. When do you think you should you stop taking antimicrobials once you've begun treatment? (When you've taken all of the antimicrobials as directed)	74.1 (372/502)	73.4 (414/564)	0.067	0.796
Q4. Will you buy the same antimicrobials, or request these from a doctor, if you're sick and they helped you get better when you had the same symptoms before? (Yes)	25.3 (127/502)	69.3 (391/564)	206.099	<0.001
Q5. Will you use antimicrobials that were given to a friend or family, as long as they were used to treat the same illness? (Yes)	28.9 (145/502)	66.8 (377/564)	153.148	<0.001

* Numbers in parentheses are the numbers of respondents vs. total participants. n: number of respondents; N: total participants.

**Table 3:** The attitude of respondents regarding antimicrobials.

**Choice question (response)**	**Strongly agree % (2022/2017)**	**Agree % (2022/2017)**	**Neutral % (2022/2017)**	**Disagree % (2022/2017)**	**Strongly disagree % (2022/2017)**	Median^*^(IQR) (2022/2017)	**Z^a^**	***P*-value**
**Positive attitude item**								
Q1. Antimicrobial resistance is one of the biggest problems the world faces.	41.2/32.3	33.3/38.3	16.3/24.6	7.8/3.0	1.4/1.8	2(2)/2(2)	−2.269	0.023
Q2. Everyone needs to take responsibility for using antimicrobials responsibly.	77.5/76.8	18.3/16.0	3.8/6.6	0.2/0.4	0.2/0.4	1(0)/1(0)	−0.533	0.594
Q3. I am worried about the impact that antimicrobial resistance will have on my health, and that of my family.	43.8/56.6	34.9/28.4	16.3/13.3	3.6/1.2	1.4/0.5	2(1)/1(1)	4.346	<0.001
Q4. Doctors should only prescribe antimicrobials when they are needed.	82.1/50.0	14.9/29.1	1.2/16.7	1.4/2.8	0.4/1.4	1(0)/2(1)	−11.399	<0.001
Q5. People should use antimicrobials only when they are prescribed by a doctor.	81.9/67.0	15.3/23.6	1.8/8.9	0.6/0	0.4/0.5	1(0)/1(1)	−5.731	<0.001
Q6. Parents should make sure all of their children's vaccinations are up-to-date.	71.9/59.6	14.9/25.4	8.2/13.7	2.8/0.9	2.2/0.5	1(1)/1(1)	−3.577	<0.001
Q7. Farmers should give fewer antimicrobials to food-producing animals.	57.0/60.5	26.7/23.8	11.8/14.5	2.0/0.4	2.6/0.9	1(1)/1(1)	1.174	0.241
Q8. People should wash their hands regularly.	90.6/80.7	6.0/11.7	2.6/6.7	0.8/0.9	0.0/0.0	1(0)/1(0)	−4.591	<0.001
**Negative attitude item**								
Q9. There is not much people like me can do to stop antimicrobial resistance.	20.9/15.6	23.1/22.7	23.1/52.8	16.7/6.6	16.1/2.3	3(2)/3(1)	2.682	0.007
Q10. I am not at risk of getting an antimicrobial resistant infection, as long as I take my antimicrobials correctly.	14.1/14.7	16.1/30.5	20.3/25.2	27.5/18.1	21.9/11.5	3(2)/3(2)	5.833	<0.001

* 1: Strongly agree; 2: Agree; 3: Neutral; 4: Disagree; 5: Strongly disagree; IQR: Interquartile range. ^a^Mann-Whitney U test.

## Results and discussion

The present study assessed the knowledge, attitudes, and practices of medical undergraduates toward AMR between 2017 and 2022. In 2017, 564 medical students responded to the poll, with 63.3 percent of them being female and a median age of 21. In 2022, the second round of surveys included 502 participants, 59.2 percent of whom were female and had a median age of 21 (Table 1).

Table 2A shows the results of the knowledge assessment. The majority of the assertions about the emergence, transmission, and detriment of antimicrobial resistance were correctly identified by respondents. However, gaps in the issues contributing to AMR, such as Q1 and Q7, were still observed in our study, indicating that the confusion regarding the consequences of antimicrobial targets was still present among these medical students. “Antimicrobial resistance occurs when your body becomes resistant to antimicrobials and they no longer work as well,” according to WHO, is a false assertion (21). Only 67.3% and 45.6% of the respondents chose the correct answer in 2022 and 2017 (*p* < 0.001), respectively, down from the prior rate of 76% published by WHO in 2016. On the other hand, 76.5% and 77.8% of the respondents considered that bacteria could be transmitted from person to person, accompanied by a proportion of students holding the opposite opinion, indicating the importance of educating the students with regard to the basic knowledge of antimicrobial resistance and bacterial transmission.

The perspectives of the respondents concerning antimicrobial use and AMR transmission are shown in Tables 2B and 3. In 2017, over two-thirds (73.4%) of respondents agreed on antimicrobial abuse in China, and 77.5% considered that antimicrobials were widely used in agriculture. However, the survey of 2022 witnessed a dramatic drop in statistics that fewer than half of the students (44.6%) believed antimicrobials were applied broadly in agriculture and food-producing animals (*p* < 0.001). Following that, particular attitudes were evaluated to illustrate the variations in antimicrobial resistance. A majority of respondents (76.8% in 2022 and 77.5% in 2017) agreed on the current abuse of antimicrobials and would like to take responsibility for reducing them. However, still 30.2 to 45.2% (strongly agree% + agree%) of the respondents firmly asserted that there is no risk of antimicrobial resistance as long as the antimicrobials are taken correctly (Table 3). Significance was observed in other attitude responses such as the impact of AMR on health, and the prescription of antimicrobials, suggesting the present problems of overconfidence and inappropriate attitudes (Table 3, *p* < 0.001).

The respondents' practice on AMR was evaluated in Table 2C. Up to 80.9% and 77.1% of respondents claimed that they received antimicrobials from doctors in 2022 and 2017, respectively. Furthermore, only a tiny proportion of participants (4.8% in 2022; 6.2% in 2017) stated they would use antimicrobials to treat the common cold, which is not recommended by WHO. In addition, around 74.1% and 73.4% expressed their willingness to take all the antimicrobials as prescribed after they started therapy, as advised by WHO. However, a considerable proportion of respondents still contemplate buying antimicrobials on their own or through a doctor if they believe they are suffering from the same condition as previously (25.3% in 2022; 69.3% in 2017, *p* < 0.001), and some even buy antimicrobials from friends or family members to treat the same illness (28.9% in 2022; 66.8% in 2017, *p* < 0.001).

For current and future stakeholders, particularly university students in the fields of medicine and medical sciences, an overall awareness of the concept of AMR as well as KAP of underlying specific concerns such as proper usage is essential. Therefore, the grading standards were enrolled to evaluate the general KAP of students between 2017 and 2022. The scores were calculated according to the grading standards and statistical test by Mann–Whitney with *p*-value < 0.001 was considered statistically significant. Significances were observed in the scores of knowledge (*p* < 0.001), practices (*p* < 0.001) and total respondents (*p* < 0.001). The present results revealed that the knowledge and practice scores had significantly improved in 2022 (*p* < 0.001), whereas there was no significance in the attitude section compared to 2017 (*p* = 0.2805, Figure 1).

In addition, we calculated the correlation of the student's knowledge, attitudes and practices among all respondents. Results showed that the students' knowledge of antimicrobials had a correlation with attitude (*p* = 0.0126) and practice (*p* < 0.001), suggesting that public education on knowledge could influence the behaviors among the medical students. Similar surveys were conducted in China and other countries focusing on public education to improve the irrational and indiscriminate use of antimicrobials in the community. The public education that provides guidelines for medical practitioners may promote the cautious and rational use of antimicrobials (15, 22).

Antimicrobial resistance was reported to be transferred through agriculture, food-producing animals, irrational prescription, and others (23–25). Therefore, utilizing the medical school's key position in public education, introducing pharmaceutical lectures, and conducting publicity outreach would effectively boost the knowledge of antimicrobial resistance in universities (22). On the other hand, there was a small proportion of the respondents who believed that they were not at risk of AMR infection. Antimicrobials, however, are responsible for more than half of all medical adverse effects, according to the China Adverse Reaction Monitoring Center (26). The increasing cases of antimicrobial-resistant bacteria caused by improper use of antimicrobials have also been observed in recent years (27). Patients may be in danger of contracting antimicrobial-resistant bacteria if they seek medications for common cold and receive them without a prescription. Inadequate dosage, incomplete treatment, and drug abuse contribute to the emergence of AMR, which is a wake-up call for us to recognize the worldwide significance of antimicrobial resistance.

Another interesting research finding is that undergraduates' awareness of antimicrobial resistance has greatly risen over the 5 years from 2017 to 2022. This finding could be attributed to the following factors; Notably, the *National Action Plan for Containment of Bacterial Resistance (2016–2020)* emphasized the necessity of enhancing the medical knowledge popularization in the universities and raising the awareness of antimicrobial resistance as well as its responsible use (28, 29). Antimicrobial resistance is being widely debated and published on social media, which may enhance public awareness among university students, in addition to the identification of the topic within the university context and its implementation into the micro curriculum (30).

This study has some limitations. First, the results of KAP among medical undergraduates in Suzhou, East China, are limited to the study population, which may cause a negative impact on the generalizability of the study findings. Second, the limited number of questions cannot adequately capture all aspects of antimicrobial resistance. Third, this study aimed to understand the general perceptions of KAP among medical undergraduates, without taking into account the direct impact of other factors such as microbiology and pharmacology courses on cognitive outcomes. Despite these limitations, the present findings provide important information for evaluating and improving the knowledge, attitude, and practice of medical undergraduate students toward antimicrobial use.

## Conclusion

We highlight those medical students surveyed had significantly increased the knowledge of antimicrobial resistance during the 5 years from 2017 to 2022 (*p* < 0.001). However, overconfident attitudes and inappropriate behaviors of AMR were still observed in the present respondents. Gaps were still observed in the issues of antimicrobial targets and bacterial transmission, indicating the importance of educating the students with regard to AMR. Additional curriculum reforms will be needed to add more specific AMR-related lectures and clinical practices to raise awareness amongst medical students in East China. The development of institutional treatment guidelines on antimicrobial resistance, and restrictions on self-prescription of unneeded antimicrobials were also urgent to reduce bacterial drug resistance in China.

## Data availability statement

The original contributions presented in the study are included in the article/Supplementary material, further inquiries can be directed to the corresponding author/s.

## Ethics statement

The studies involving human participants were reviewed and approved by Ethics Committees of Suzhou Medical College in Jiangsu Province, China. The patients/participants provided their written informed consent to participate in this study.

## Author contributions

SM, HL, ZZ, and HZ wrote the main manuscript text. SM, YZ, YS, and JY prepared the tables. YD and XW analyzed the statistical data. All authors reviewed the manuscript.

## Funding

This work was supported by grants from Extracurricular Scientific Research Project for Students of Suzhou Medical College (Pasteurien College), and Suzhou Science and Technology Project (N316460121).

## Conflict of interest

The authors declare that the research was conducted in the absence of any commercial or financial relationships that could be construed as a potential conflict of interest.

## Publisher's note

All claims expressed in this article are solely those of the authors and do not necessarily represent those of their affiliated organizations, or those of the publisher, the editors and the reviewers. Any product that may be evaluated in this article, or claim that may be made by its manufacturer, is not guaranteed or endorsed by the publisher.
