# Supplementary material for: Knowledge, attitude, and practice associated with antimicrobial resistance among medical students between 2017 and 2022: A survey in East China
Source: Front Public Health. 2022 Oct 24;10:1010582. doi: 10.3389/fpubh.2022.1010582 (PMC9637849; doi:10.3389/fpubh.2022.1010582)
Supplement: Supplementary file 1 [file Table_1.DOCX]

**Supply material 1. Questionnaire**

**First section**

**Gender:**

**Age:**

**Education:**

**In this survey we would like to know how medical undergraduates think about antibiotic resistance including the parts of attitude, knowledge and practice.**

**Second section**

**1) Do you think these conditions can be treated with antimicrobials? (Multiple choices)**

1. HIV/AIDS

2. Gonorrhoea

3. Bladder infection or urinary tract infection (UTI)

4. Diarrhoea

5. Cold and flu

6. Fever

7. Malaria

8. Measles

9. Skin or wound infection

10. Sore throat

11. Body aches

12. Headaches

**2) Have you heard of any of the following terms? (Multiple choices)**

1. Antibiotic resistance

2. Superbugs

3. Antimicrobial resistance

4. AMR

5. Drug resistance

6. Antibiotic-resistant bacteria

**Third section**

**3) Please indicate whether you think the following statements are ‘true’ or ‘false’**

1. Antibiotic resistance occurs when your body becomes resistant to antimicrobials and they no longer work as well.

2. Many infections are becoming increasingly resistant to treatment by antimicrobials.

3. If bacteria are resistant to antimicrobials, it can be very difficult or impossible to treat the infections they cause.

4. Antibiotic resistance is an issue that could affect me or my family.

5. Antibiotic resistance is an issue in other countries but not here.

6. Antibiotic resistance is only a problem for people who take antimicrobials regularly.

7. Bacteria which are resistant to antimicrobials can be spread from person to person.

8. Antibiotic-resistant infections could make medical procedures like surgery, organ transplants and cancer treatment much more dangerous.

**Forth section**

**4) Do you think there is abuse of the current antimicrobials?**

1. Yes 2. No 3. Don’t know

**5) Do you think antimicrobials are widely used in agriculture (including in food-producing animals) in your country?**

1. Yes 2. No 3. Don’t know

**6) On the scale shown, how much do you agree with following statements?**

|  | **Agree strongly** | **Agree slightly** | **Neither agree nor disagree** | **Disagree slightly** | **Disagree strongly** |
| --- | --- | --- | --- | --- | --- |
| **1. Antibiotic resistance is one of the biggest problems the world faces.** | 5 | 4 | 3 | 2 | 1 |
| **2. Everyone needs to take responsibility for using antimicrobials responsibly.** | 5 | 4 | 3 | 2 | 1 |
| **3. I am worried about the impact that antibiotic resistance will have on my health, and that of my family.** | 5 | 4 | 3 | 2 | 1 |
| **4. Doctors should only prescribe antimicrobials when they are needed.** | 5 | 4 | 3 | 2 | 1 |
| **5. People should use antimicrobials only when they are prescribed by a doctor.** | 5 | 4 | 3 | 2 | 1 |
| **6. Parents should make sure all of their children’s vaccinations are up-to-date.** | 5 | 4 | 3 | 2 | 1 |
| **7. Farmers should give fewer antimicrobials to food-producing animals.** | 5 | 4 | 3 | 2 | 1 |
| **8. People should wash their hands regularly.** | 5 | 4 | 3 | 2 | 1 |
| **9. There is not much people like me can do to stop antibiotic resistance.** | 5 | 4 | 3 | 2 | 1 |
| **10. I am not at risk of getting an antibiotic resistant infection, as long as I take my antimicrobials correctly.** | 5 | 4 | 3 | 2 | 1 |

**Fifth section**

**7) Where do the antimicrobials you used come from?**

1. Doctor or nurse (Pharmacist)

2. Family member

3. Friend (including on social media)

4. Buy them by yourself

5. other

**8) Will you ask the doctor to prescribe for you antimicrobials when you catch a common cold?**

1. Yes 2. No 3. Don’t know

**9) When do you think you should stop taking antimicrobials once you’ve begun treatment?**

1. When you feel better

2. When you’ve taken all of the antimicrobials as directed

3. Don’t know

**10) Will you buy the same antimicrobials, or request these from a doctor, if you’re sick and they helped you get better when you had the same symptoms before?**

1. Yes 2. No 3. Don’t know

**11) Will you use antimicrobials that were given to a friend or family member, as long as they were used to treat the same illness?**

1. Yes 2. No 3. Don’t know
